# Associations of ultra-processed food consumption with cardiovascular disease and all-cause mortality: UK Biobank

**DOI:** 10.1093/eurpub/ckac104

**Published:** 2022-08-25

**Authors:** Xuanli Chen, Jiadong Chu, Wei Hu, Na Sun, Qida He, Siyuan Liu, Zhaolong Feng, Tongxing Li, Qiang Han, Yueping Shen

**Affiliations:** Department of Epidemiology and Biostatistics, School of Public Health, Medical College of Soochow University, Suzhou 215123, China; Department of Epidemiology and Biostatistics, School of Public Health, Medical College of Soochow University, Suzhou 215123, China; Department of Epidemiology and Biostatistics, School of Public Health, Medical College of Soochow University, Suzhou 215123, China; Department of Epidemiology and Biostatistics, School of Public Health, Medical College of Soochow University, Suzhou 215123, China; Department of Epidemiology and Biostatistics, School of Public Health, Medical College of Soochow University, Suzhou 215123, China; Department of Epidemiology and Biostatistics, School of Public Health, Medical College of Soochow University, Suzhou 215123, China; Department of Epidemiology and Biostatistics, School of Public Health, Medical College of Soochow University, Suzhou 215123, China; Department of Epidemiology and Biostatistics, School of Public Health, Medical College of Soochow University, Suzhou 215123, China; Department of Epidemiology and Biostatistics, School of Public Health, Medical College of Soochow University, Suzhou 215123, China; Department of Epidemiology and Biostatistics, School of Public Health, Medical College of Soochow University, Suzhou 215123, China

## Abstract

**Background:**

This study aimed to investigate the associations between ultra-processed food (UPF) consumption and the risk of cardiovascular disease and all-cause mortality in the UK Biobank Cohort.

**Methods:**

This observational prospective study evaluated 60 298 participants aged 40 years or older. We used the NOVA classification system to identify and categorize UPF. The associations among UPF consumption, cardiovascular disease (CVD) incidence and all-cause mortality were estimated using multivariable Cox proportional hazards models. Dose–response analysis of UPF consumption and CVD incidence and mortality was performed using a restricted cubic spline.

**Results:**

After a median follow-up of 10.9 years, 6048 participants (10.0%) experienced CVD events, and 5327 (8.8%) and 1503 (2.5%) experienced coronary heart and cerebrovascular diseases, respectively. There were 2590 (4.3%) deaths, of which 384 (0.6%) deaths were caused by CVD. A higher intake of UPF was associated with a higher risk of CVD and all-cause mortality (all *P *<* *0.001). A higher intake of UPF was associated with a higher risk of CVD [hazard ratio (HR) = 1.17, 95% confidence interval (CI): 1.09–1.26], coronary heart disease (HR = 1.16, 95% CI: 1.07–1.25), cerebrovascular disease (HR = 1.30, 95% CI: 1.13–1.50) and all-cause mortality (HR = 1.22, 95% CI: 1.09–1.36). The association of UPF consumption with a range of CVD incidents and all-cause mortality was monotonic (all *P* for non-linearity > 0.30).

**Conclusions:**

A higher proportion of UPF consumption was associated with CVD and all-cause mortality. Thus, actions to limit UPF consumption should be incorporated into the CVD and all-cause mortality prevention recommendations.

## Introduction

Cardiovascular disease (CVD) is a notable cause of morbidity and mortality among non-communicable diseases worldwide.^1^ Dietary habits influence many risk factors of cardiometabolic health, stroke and heart disease. A healthy and balanced diet can reduce the risk of CVD by ∼50%.^2^ The NOVA system classifies all food products into four groups according to the processing techniques they undergo.^3^^,^^4^ Ultra-processed food (UPF), including breakfast cereals, sweets, soft drinks, sausages etc.,^4^ classified in Group IV of the NOVA system,^3^ account for more than half of food consumption in many developed countries,^5–8^ representing between 25% and 60% of the total daily energy. Most UPF contain industrial formulation substances and classes of additives and involve processing techniques that may lead to the production and introduction of compounds with potentially cardiometabolic effects. UPF often contain preservatives for longer shelf lives, which facilitate the potential migration of materials in contact with food. Evidence of the relationship between the degree of food processing and health outcomes,^9^ such as overweight/obesity, elevated waist circumference, reduced HDL-cholesterol levels, increased risk of metabolic syndrome, all-cause mortality, CVD, cerebrovascular disease, type 2 diabetes, cancer and depression,^9–11^ has grown rapidly over the decade.

However, few studies have investigated the role of UPF in CVD risk,^12–14^ and there are inconsistent results regarding CVD mortality in prospective studies.^15^^,^^16^ In a prospective cohort within the Moli-Sani study, UPF intake was associated with a higher risk of CVD mortality over a median follow-up period of 8.2 years. Further elucidation of the association between UPF levels and CVD mortality is crucial in large cohort studies. Given the high levels of UPF consumption and high economic burden of CVD in the UK, this study aimed to explore the association of UPF with CVD, all-cause mortality and CVD mortality. Towards this goal, we analysed data from a large sample of the British Biobank (2006–2020).

## Methods

### Study design and population

UK Biobank, a large ongoing prospective cohort study, started collecting health information from half a million UK citizens aged 37–73 years in 2007^17^ and it is an open access data resource for researchers with academic ethics requirements (https://www.ukbiobank.ac.uk/, 19 August 2022, date last accessed). At baseline, participants provided informed consent and completed a self-administered, touch screen questionnaire and face-to-face interviews in 22 assessment centres across England, Scotland and Wales. The participants’ National Health Service (NHS) health information was regularly updated in each follow-up assessment. The CVD and mortality data were updated on 31 December 2020.

### Exposure ascertainment

Participants were invited to complete a web-based 24-h dietary recall questionnaire developed by Oxford WebQ (www.ceu.ox.ac.uk/research/oxford-webq, 19 August 2022, date last accessed). The 24-h dietary recall questionnaire was used to investigate the consumption of 204 common food and drink items during the previous 24 h,^18^ which had five cycles in all (Supplementary figure S1). Between 2009 and 2010, cycle 0 included a 24-h recall questionnaire administered by the interviewer at the assessment centre, and the remaining four cycles of the questionnaire were completed by sending a web link to the participants who had provided an email address every 3–4 months.^19^ The interviewer-administered 24-h dietary recall was more representative of their dietary intake at baseline, the current analysis used data collected in cycle 0.

UPF consumption was estimated based on the interviewer-administered 24-h dietary recall of each participant in cycle 0. All food and drink items were classified according to the NOVA food classification system.^3^ The food item classified as the Group IV of the NOVA system (UPFs group) in the 24-h dietary recall questionnaire was shown in Supplementary table S1.

We assigned typical serving sizes and appropriate total energy supply per 100 g or 100 ml to each food and beverage item to derive our own estimated energy intake values based on published UK data.^20^^,^^21^ Then, the energy intake contributed by the UPFs group (57 food items) and the non-UPF group was calculated for each individual. This study primarily focussed on the UPF group (see Supplementary appendix S1 for details about the calculation). The energy contribution of UPFs can be calculated by the following formula:

$$E_{c}=\frac{E_{\mathrm{UPFs}}}{E_{\mathrm{UPFs}}+E_{\mathrm{non}-\mathrm{UPFs}}}$$

where $E_{\mathrm{UPFs}}$ is the energy intakes of UPF group, $E_{\mathrm{non}-\mathrm{UPFs}}$ is the energy intakes of non-UPFs group, and $E_{c}$ is the energy contribution of UPFs in total energy. Furthermore, we classified $E_{c}$ into four groups according to quartiles.

### Outcome ascertainment

We defined CVD as angina pectoris, acute and subsequent myocardial infarction, certain current complications following acute myocardial infarction, other acute ischaemic heart diseases, chronic ischaemic heart disease, atrial fibrillation and flutter, heart failure, subarachnoid haemorrhage, intracerebral haemorrhage, other non-traumatic intracranial haemorrhage, cerebral infarction, stroke, not specified as haemorrhage or infarction, other cerebrovascular diseases, cerebrovascular disorders in diseases classified elsewhere, and sequelae of cerebrovascular disease. The corresponding International Classification of Diseases, 10th revision (ICD-10) codes for CVD are I20–I25, I48, I50, I60–I64 and I67–I69.The ICD-10 codes for coronary heart disease (CHD) are I20–I25, I48 and I50, and the ICD-10 codes for cerebrovascular disease are I60–I64 and I67–I69.^22^ Person years for each participant were calculated from baseline until the date when the outcome was identified, lost to follow-up, or last follow-up, whichever occurred first. The last event was ascertained on the last follow-up date (31 December 2020).

Death, cause of death and incident CVD cases were obtained through linkage to the NHS Information Centre, NHS Central Register Scotland, Hospital Episode Statistics-Admitted Patient Care (England), Scottish Morbidity Records-General/Acute Inpatient and Day Case Admissions (Scotland), and patient episode database to 31 December 2020.

### Evaluation of covariates

The participants underwent a series of standardized anthropometry and blood assays at baseline, including height, weight, blood pressure, total cholesterol, low-density lipoprotein cholesterol (LDL-C), high-density lipoprotein cholesterol (HDL-C) and glucose. Age, sex, years of education, sleep duration and smoking status were self-reported, as well as the health status of hypertension, dyslipidaemia and diabetes. The Townsend deprivation index (TDI) score, which was derived from unemployment, car and homeownership, and overcrowding, was assigned to each participant corresponding to their area postcode. Physical activity level was assessed using the total metabolic equivalent task by using guidelines for data processing and analysis of the International Physical Activity Questionnaire. Total energy, protein, total fat, carbohydrates, alcohol, fibre, saturated fat, polyunsaturated fat and trans-fat intakes were calculated using a 24-h dietary recall questionnaire. The details of the UK Biobank field IDs and the corresponding variables involved in this study can be found in Supplementary table S2.

### Statistical analysis

To avoid imputing covariates for a massive number of participants or excluding those with missing covariate data, we adopted the following strategies: if <5%, the missing values were imputed by the modal value for categorical variables or the median for continuous variables; if more than 5%, they were dealt with as missing values. The median (lower quartile-upper quartile), mean (mean ± standard deviation) and frequency (percentage) were used to describe continuous and categorical variables. Baseline characteristics were described across different levels of UPFs, and group differences by quartiles of UPFs were evaluated using the Pearson chi-square test, Kruskal–Wallis test or analysis of variance as appropriate.

The shape of the cumulative risk of incidence of CVD and all-cause mortality was examined using Kaplan–Meier plot curves and the log-rank test according to quartiles of UPF consumption. Using the lowest quartile as a reference category, multivariable Cox proportional hazards regression was performed to estimate the hazard ratio (HR) and 95% confidence intervals (95% CIs) between exposure to UPF consumption and the risk of CVD and all-cause mortality. The proportional assumption of the Cox proportional hazards models was tested using the Schoenfeld residual. Model 1 included quartiles of UPF consumption, age, sex, ethnicity, years of education, smoking status and TDI. Model 2 was additionally adjusted for obesity status, sleep duration, total energy intake, physical activity, protein, total fat, carbohydrates, alcohol, fibre, saturated fat, monounsaturated fat, polyunsaturated fat and trans-fat. Model 3 was based on Model 2 and was additionally adjusted for hypertension, diabetes and dyslipidaemia. The associations of UPF consumption in each group with the risk of CHD, cerebrovascular diseases, and death caused by CVD was additionally analysed to determine whether the associations were driven by a specific subcategory of CVD or CVD. The assumption of linearity between UPF consumption and the risk of these health outcomes was verified by using restricted cubic spline functions with the RMS package (five knots).

To test potential variations in different subgroups and the robustness, we also investigated the relationship between the consumption of UPFs and the risk of CVD in each subgroup: male and female, younger (<60 years) and older (≥60 years), participants with BMI <25 kg/m^2^ and those with BMI ≥25 kg/m^2^, participants with lower TDI and those with higher TDI. Several sensitivity analyses were performed as follows: (i) events diagnosed during the first 2 years of each participant’s follow-up were excluded; (ii) participants with web-based 24-h dietary recall questionnaire were merged; and (iii) participants whose proportion of UPFs in the diet varied by <|0.2| between any two cycles of their 24-h dietary recall questionnaire.

All statistical analyses were performed using SAS version 9.4 and R version 4.0.5. The significance level was set at a two-sided value of 0.05.

## Results

### Characteristics of the participants

A total of 60 298 participants (55.6% women) were included in this study (Supplementary figure S2). The median age at baseline was 57 (49–62) years. Some characteristics were excluded because missing 24-h diet questionnaire data were statistically significant, but not clinically significant, compared with those at baseline in this study (Supplementary table S3). Table 1 shows the baseline characteristics of the participants according to the quartiles of dietary UPF consumption. Compared with participants in the lowest quartile, participants in the highest quartile of UPF intake showed a higher proportion of participants with younger age, male sex, white race, current smokers, higher BMI, higher total energy, higher total fat, higher carbohydrate, higher carbohydrate, higher saturated fat, higher polyunsaturated fat and higher trans-fat intake. Meanwhile, who had lower TDI, education years, sleep duration, physical activity, protein, alcohol and fibre intake.

**Table 1:** Distributions of characteristics of the study population at baseline stratified by quartile of consumption of ultra-processed foods (2008) in UK Biobank

Median (low quartile-upper quartile) or mean ± standard deviation or quantity (percentage)						
Quartile^a^ of ultra-processed foods consumption (% of total energy)						
Variables	All participants	Group 1	Group 2	Group 3	Group 4	*P* value^b^
*N*	60 298	15 075 (25.00)	15 073 (25.00)	15 075 (25.00)	15 075 (25.00)	
Age, years	57 (49–62)	57 (50–62)	57 (49–63)	57 (49–62)	55 (48–62)	<0.001
Sex (Male)	26 767 (44.39)	5995 (39.77)	6485 (43.02)	6920 (45.90)	7367 (48.87)	<0.001
Education levels, years						
≤10	22 167 (36.76)	4617 (30.63)	5250 (34.83)	5662 (37.56)	6638 (44.03)	<0.001
10 to ≤18	10 693 (17.73)	2632 (17.46)	2731 (18.12)	2747 (18.22)	2583 (17.13)	
>18	27 438 (45.50)	7826 (51.91)	7092 (47.05)	6666 (44.22)	5854 (38.83)	
Ethnicity (White)	56 305 (93.38)	13 801 (91.55)	14 071 (93.35)	14 205 (94.23)	14 228 (94.38)	<0.001
Townsend index (lower)	18 356 (30.44)	4277 (28.37)	4662 (30.93)	4833 (32.06)	4584 (30.41)	<0.001
Sleep duration, h						
<7	14 636 (24.27)	3623 (24.03)	3548 (23.54)	3576 (23.72)	3889 (25.80)	<0.001
7 to ≤8	41 907 (69.50)	10 523 (69.80)	10 619 (70.45)	10 561 (70.06)	10 204 (67.67)	
>8	3755 (6.23)	929 (6.16)	906 (6.01)	938 (6.22)	982 (6.51)	
Physical activity						
Lower tertiles	15 707 (26.05)	3733 (24.76)	3878 (25.73)	3957 (26.25)	4139 (27.46)	<0.001
Middle	17 555 (29.11)	4650 (30.85)	4483 (29.74)	4365 (28.96)	4057 (26.91)	
Upper tertiles	17 344 (28.76)	4502 (29.86)	4368 (28.98)	4252 (28.21)	4222 (28.01)	
Missing	9692 (16.07)	2190 (14.53)	2344 (15.55)	2501 (16.59)	2657 (17.63)	
Smoking status						
Never	34 974 (58.00)	8297 (55.04)	8833 (58.60)	8961 (59.44)	8883 (58.93)	<0.001
Ever	20 214 (33.52)	5512 (36.56)	5070 (33.64)	4929 (32.70)	4703 (31.20)	
Current	5110 (8.47)	1266 (8.40)	1170 (7.76)	1185 (7.86)	1489 (9.88)	
BMI status, kg/m^2^						
Underweight (<18.5)	186 (0.31)	64 (0.42)	45 (0.30)	40 (0.27)	37 (0.25)	<0.001
Normal weight (18.5 to < 25)	21 718 (36.02)	5852 (38.82)	5541 (36.76)	5396 (35.79)	4929 (32.70)	
Overweight (25 to <30)	25 131 (41.68)	6170 (40.93)	6278 (41.70)	6324 (41.95)	6350 (42.12)	
Obese (≥30)	13 263 (22.00)	2989 (19.83)	3200 (21.23)	3315 (21.99)	3759 (24.94)	
Total energy, kJ/d						
Lower tertiles	20 912 (34.68)	6227 (41.31)	5095 (33.80)	4692 (31.12)	4898 (32.49)	<0.001
Middle	19 228 (31.89)	4767 (32.87)	4954 (32.87)	4892 (32.45)	4615 (30.61)	
Upper tertiles	20 158 (33.43)	4081 (27.07)	5024 (33.33)	5491 (36.42)	5562 (36.90)	
Protein, g/day	81.7 ± 31.0	83.8 ± 32.2	85.5 ± 31.1	83.3 ± 30.3	74.9 ± 29.5	<0.001
Total fat, g/day	76.9 ± 36.2	69.2 ± 35.0	76.5 ± 36.1	80.0 ± 36.4	80.7 ± 36.2	<0.001
Carbohydrates, g/day	257.9 ± 108.1	232.7 ± 97.9	256.4 ± 103.0	268.3 ± 108.9	270.6 ± 115.9	<0.001
Alcohol, g/day	15.8 ± 24.0	23.0 ± 29.0	18.4 ± 24.9	14.4 ± 22.1	8.8 ± 17.2	<0.001
Fibre, g/day	16.6 ± 7.8	16.8 ± 8.6	17.5 ± 7.9	17.0 ± 7.5	15.2 ± 7.1	<0.001
Saturated fat, g/day	29.6 ± 15.8	24.8 ± 13.9	29.0 ± 15.2	31.1 ± 16.1	32.6 ± 17.0	<0.001
Polyunsaturated fat, g/day	14.2 ± 8.8	13.7 ± 9.3	14.6 ± 9.1	14.7 ± 8.8	13.9 ± 8.1	<0.001
Trans-fat, g/day	3.1 ± 6.6	1.6 ± 1.7	2.5 ± 3.5	4.2 ± 8.9	3.9 ± 8.4	<0.001
Hypertension (yes)	29 488 (48.90)	7343 (48.71)	7453 (49.45)	7421 (49.23)	7271 (48.23)	0.364
Diabetes (yes)	1324 (2.20)	312 (2.07)	343 (2.28)	328 (2.18)	341 (2.26)	0.371
Dyslipidaemia (yes)	31 942 (52.97)	7833 (51.96)	8052 (53.42)	8049 (53.39)	8008 (53.12)	0.057

a Group 1 is the proportion UPFs energy of total energy in Quartile 1; Group 2 is the proportion UPFs energy of total energy Quartile 2; Group 3 is the proportion UPFs energy of total energy in Quartile 3; Group 4 is the proportion UPFs energy of total energy in Quartile 4. The cut-off values for quarters of ultra-processed food consumption were 20.8%, 31.3%, 43.0% for all participants.

b Analysis of variance, Kruskal–Wallis test or *χ*^2^ test where appropriate.

### Associations of UPF with CVD, CHD, cerebrovascular disease, all-cause mortality and CVD mortality risk in the different model

**Figure 1:**
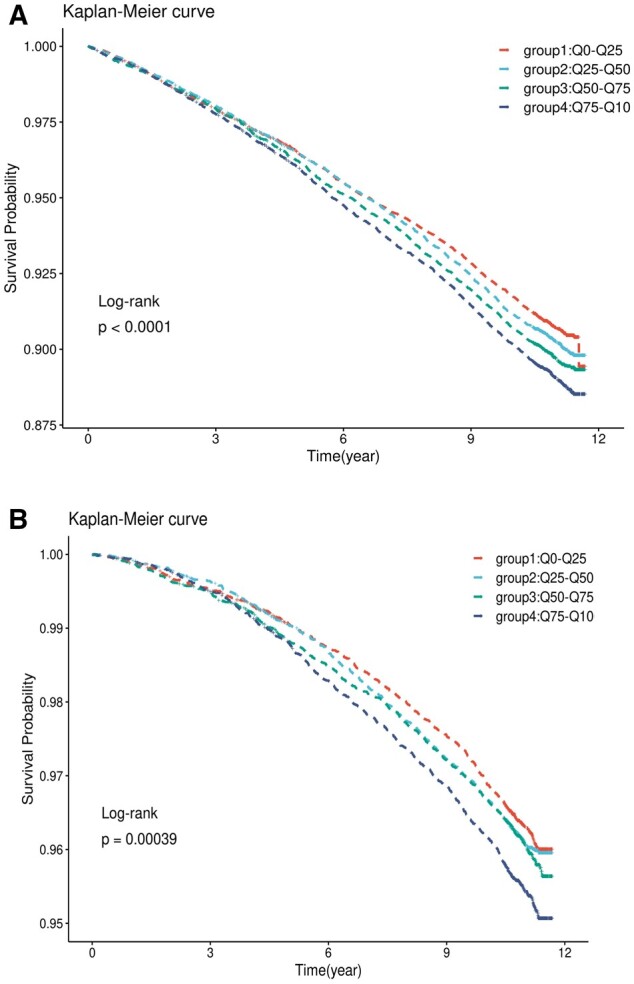
Kaplan–Meier curves for the cumulative risk of CVD (left) and all-cause mortality (right) in four groups according to consumption of ultra-processed foods (2008-2020) in UK Biobank

After a median follow-up period of 10.9 years, the person–year incidences of CVD in groups 1, 2, 3 and 4 were 8.79‰, 9.26‰, 9.79‰ and 10.48‰, respectively (Table 2). The person–year incidences of CHD, cerebrovascular disease, all-cause mortality, and CVD mortality in all groups are shown in Table 2. The survival curves of new-onset CVD according to the quartiles of UPF consumption were significantly different in the Kaplan–Meier analysis (log-rank *P *<* *0.001). Similar results were observed for the all-cause mortality. Group 4 (highest quartiles of UPF consumption) had the highest risk of new-onset CVD and all-cause mortality (figure 1).

**Table 2:** The association between ultra-processed foods intake and CVD, CHD, cerebrovascular disease and CVD mortality according to consumption of ultra-processed foods (2008–20) in UK Biobank

	Group 1	Group 2	Group 3	Group 4	*P* trend
**New-onset CVD**					
Cases^a^ [*n* (%)]	1390 (9.22)	1466 (9.73)	1544 (10.24)	1648 (10.93)	
Person-year incidence	8.79‰	9.26‰	9.79‰	10.48‰	
Unadjusted	1 (ref)	1.07 (1.00–1.15)	1.10 (1.03–1.18)	1.17 (1.09–1.25)	<0.001
Model 1^b^	1 (ref)	1.06 (0.98–1.13)	1.08 (1.01–1.16)	1.18 (1.10–1.26)	<0.001
Model 2^c^	1 (ref)	1.05 (0.98–1.13)	1.08 (1.00–1.16)	1.16 (1.08–1.25)	<0.001
Model 3^d^	1 (ref)	1.05 (0.97–1.12)	1.07 (0.99–1.15)	1.15 (1.07–1.24)	<0.001
**New-onset CHD**					
Cases^a^ [*n* (%)]	1218 (8.00)	1293 (8.49)	1369 (8.99)	1447 (9.50)	
Person-year incidence	7.58‰	8.05‰	8.55‰	9.05‰	
Unadjusted	1 (ref)	1.06 (0.98–1.15)	1.13 (1.04–1.22)	1.20 (1.11–1.29)	<0.001
Model 1^b^	1 (ref)	1.04 (0.96–1.13)	1.10 (1.02–1.19)	1.18 (1.10–1.28)	<0.001
Model 2^c^	1 (ref)	1.04 (0.96–1.13)	1.11 (1.02–1.20)	1.19 (1.09–1.26)	<0.001
Model 3^d^	1 (ref)	1.03 (0.96–1.12)	1.10 (1.01–1.19)	1.18 (1.08–1.28)	<0.001
**New-onset cerebrovascular disease**					
Cases^a^ [*n* (%)]	348 (2.19)	370 (2.33)	358 (2.25)	443 (2.78)	
Person-year incidence	2.02‰	2.14‰	2.07‰	2.57‰	
Unadjusted	1 (ref)	1.06 (0.92–1.23)	1.03 (0.89–1.19)	1.27 (1.11–1.47)	<0.001
Model 1^b^	1 (ref)	1.06 (0.91–1.20)	1.03 (0.89–1.20)	1.30 (1.13–1.50)	<0.001
Model 2^c^	1 (ref)	1.06 (0.91–1.23)	1.03 (0.88–1.20)	1.30 (1.11–1.52)	<0.001
Model 3^d^	1 (ref)	1.05 (0.91–1.22)	1.02 (0.88–1.19)	1.29 (1.10–1.51)	<0.001
**All-cause mortality**	Group 1	Group 2	Group 3	Group 4	
Cases^a^ [*n* (%)]	596 (3.71)	621 (3.85)	636 (3.95)	737 (4.57)	
Person-year incidence	3.42‰	3.56‰	3.65‰	4.24‰	
Unadjusted	1 (ref)	1.04 (0.93–1.17)	1.07 (0.95–1.19)	1.24 (1.11–1.38)	<0.001
Model 1^b^	1 (ref)	1.04 (0.93–1.16)	1.06 (0.95–1.18)	1.23 (1.10–1.37)	<0.001
Model 2^c^	1 (ref)	1.04 (0.93–1.16)	1.06 (0.95–1.19)	1.23 (1.09–1.39)	<0.001
Model 3^d^	1 (ref)	1.03 (0.92–1.16)	1.06 (0.94–1.19)	1.22 (1.08–1.38)	<0.001
**New-onset CVD mortality**					
Cases^a^ [*n* (%)]	79 (0.49)	98 (0.61)	106 (0.66)	101 (0.63)	
Person-year incidence	0.45‰	0.56‰	0.60‰	0.57‰	
Unadjusted	1 (ref)	1.24 (0.92–1.67)	1.34 (1.00–1.79)	1.28 (0.95–1.71)	0.232
Model 1^b^	1 (ref)	1.22 (0.91–1.64)	1.31 (0.98–1.75)	1.21 (0.90–1.63)	0.372
Model 2^c^	1 (ref)	1.18 (0.87–1.59)	1.24 (0.92–1.68)	1.10 (0.79–1.53)	0.383
Model 3^d^	1 (ref)	1.16 (0.86–1.57)	1.23 (0.91–1.66)	1.07 (0.77–1.49)	0.350

Values were presented as hazard ratios (95% confidence interval).

Group 1 is the proportion UPFs energy of total energy in Quartile 1; Group 2 is the proportion UPFs energy of total energy Quartile 2; Group 3 is the proportion UPFs energy of total energy in Quartile 3; Group 4 is the proportion UPFs energy of total energy in Quartile 4. The cut-off values for quarters of ultra-processed food consumption were 20.8%, 31.3%, 43.0% for all participants.

a Participants in cycle 0.

b Model 1: age, sex, ethnicity, education years, smoking status and Townsend deprivation index.

c Model 2: Model 1 with obesity status, sleep duration, total energy intake, physical activity, protein, total fat, carbohydrates, alcohol, fibre, saturated fat, monounsaturated fat, polyunsaturated fat, trans-fat.

d Model 3: Model 2 with hypertension, diabetes and dyslipidaemia.

**Figure 2:**
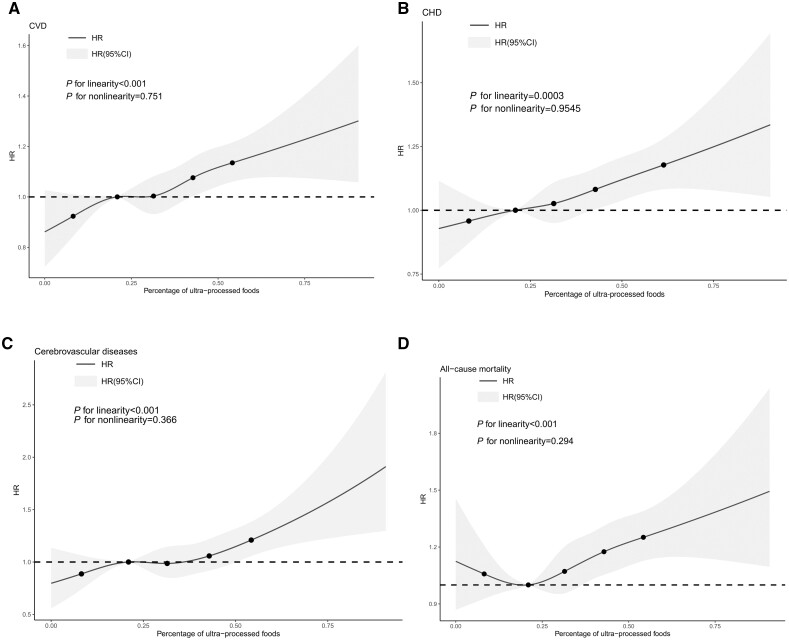
Spline plot for linearity assumption of association between proportion of UPFs in diet and risks of CVD, CHD, cerebrovascular disease and all-cause mortality according to consumption of ultra-processed foods (2008-2020) in UK Biobank

Based on the fully adjusted final model (Model 3), compared with those in the lowest quartile of consumption group, individuals with the highest quartile of consumption had a significantly higher risk of new-onset CVD (HR = 1.17, 95% CI: 1.09–1.26). Similar evidences were further found in CHD (HR = 1.16, 95% CI: 1.07–1.25), cerebrovascular disease (HR = 1.30, 95% CI: 1.13–1.50) and all-cause mortality (HR = 1.22, 95% CI: 1.09–1.36). The linearity assumptions between intake of UPF and risks of CVD, CHD, cerebrovascular disease and all-cause mortality were confirmed by the restricted cubic spline (*P* for non-linearity > 0.05, *P* for linearity < 0.05, figure 2).

### Sensitivity and subgroup analysis

Sensitivity analysis (Supplementary tables S4 and S5) focussed on the associations between the total energy contribution of UPF and indicators of health outcomes after excluding individual CVD cases diagnosed during the first 2 years of each participant’s follow-up or merging the web-based 24-h dietary recall questionnaire. Based on the fully adjusted final model (Model 3), the positive associations of UPF intake with CVD, CHD, cerebrovascular disease and all-cause mortality were significant (*P *<* *0.001). A positive association was also found for CVD mortality after including participants in the web dietary questionnaire (>180 000 participants, HR = 1.34, 95% CI: 1.13–1.60, Supplementary table S5). Sensitivity analysis (Supplementary table S6) focussed on more than 50 000 participants who had received a minimum of two 24-h dietary recall questionnaires with a change in consumption of UPFs in their diet of <|0.2|, and it provided similar results in CVD, CHD, cerebrovascular disease and all-cause mortality (*P *<* *0.001).

In all subgroups, individuals in the highest quartile of UPF consumption had the highest risk of new cases of CVD after adjusting for health behaviours, demographics, socioeconomic confounders and other clinical disease factors (Supplementary figure S3). The strength of the association between higher UPF intake and incident CVD decreased with age [HR: 1.19 (95% CI: 1.05–1.34) for adults aged <45 years and 1.13 (95% CI: 1.04–1.24) for adults aged ≥45 years]. The association between higher UPF intake and incident CVD was stronger in male participants [HR: 1.21 (95% CI: 1.08–1.36) vs. 1.15 (95% CI: 1.05–1.26)].

## Discussion

This large prospective cohort study of British adults found that the significant associations between higher UPF consumption in diet and higher risk of incident new-onset CVD, new-onset CHD, new-onset cerebrovascular diseases and all-cause mortality. This association remained significant in our sensitivity analysis.

Our findings on the relationship of UPF with CVD, CHD and cerebrovascular disease are consistent with those from the Framingham Offspring^14^ and the French NutriNet-Santé cohort studies.^23^ A similar result between the proportion of UPFs in the diet and all-cause mortality was also observed in the Moli-Sani,^12^ SUN^24^ and NHANES III cohort studies.^16^ Higher UPF intake was not associated with CVD mortality in the main results. However, a significant positive association between higher UPF intake and CVD mortality was observed in the analysis merging other cycles. The discrepant results between analyses or studies may be attributed to the Mediterranean diet,^12^^,^^25^ increased sample size and differences in dietary habits over time for each participant. The positive association between higher UPF consumption and health outcomes, where adverse health outcomes include multiple chronic diseases (especially CVD and cancer that can cause premature death), may explain the relationship between UPF consumption and all-cause mortality.

Several hypotheses could plausibly explain the observed associations. First, UPF generally have poorer nutritional quality,^3^^,^^6^^,^^8^^,^^26^ and often exhibit a richer glycaemic response with lower satiety potential.^27^ Among these determinants, excessive energy, fat and sugar contribute to weight gain and the risk of being overweight or obese as major risk factors for CVDs. Some of these nutritional compounds are known risk factors for cardiovascular health problems.^28^ For instance, sweetened beverages and processed meats in the UPF group are well-loved in Western-type diets; these foods might contain relatively high levels of advanced glycation end products that over time could lead to or accelerate cardiovascular events.^29^ In the current study, UPF intake was significantly associated with a range of CVD and mortality outcomes even after adjustment for BMI, diet quality and total intake of energy with low changes in HR. Therefore, it is reasonable to assume that these factors did not fully explain the observed associations. Processing and other bioactive compounds specifically contained in UPFs may contribute to these associations.

Secondly, UPFs contain a wide range of additives for taste and long shelf life. Although the regulated levels of food additives normally protect consumers against adverse effects in a given food product, the long-term health impacts of cumulative intake and the potential interaction effects in all ingested foods remain largely unknown.^30^ Furthermore, toxicity studies of single authorized additives are generally performed using animal or cellular models. Additives may also have toxic effects. For instance, emulsifiers (thickening agents), a common ingredient in UPFs, are associated with the development of numerous chronic inflammatory diseases in mice.^31^ A high dose of monosodium glutamate accelerates the atherosclerotic process and other CHDs through lipid peroxidation.^32^ Dietary emulsifiers alter microbiota composition and gut inflammation in mice.^33^ Overall, additives in UPF may promote a proinflammatory status and metabolic dysregulation.

Thirdly, food processing can alter the nutritional, physical and chemical characteristics of foods in ways that may alter their healthfulness, and also influence long-term dietary behaviours, satiety signalling and food reward systems. The National Health and Nutrition Examination Survey (NHANES)^34^ and the Louisville Healthy Heart Study^35^ reported that heat treatments during UPF processing produce acrylamide, a newly formed contaminant that is associated with a higher risk of CVD. Lastly, great efforts have recently been made to identify the migration process and the health and ecological hazards of plastic packaging.^36^ Several chemical compounds, including bisphenol A (BPA) and microplastics, are released from packaging materials in plastic packaging and plastic cups. BPA exposure was found to be associated with an increased risk of CVD outcomes (particularly in hypertension and coronary artery disease) using a National Health and Nutrition Examination Survey and meta-analysis.^37^ The biological pathways by which UPFs affect cardiovascular health may involve complex mechanisms and synergistic effects between many compounds and the characteristics of UPFs. Many factors, such as metabolic, pro-inflammatory, pro-thrombotic, pro-oxidative and endothelial dysfunction, are involved in the progression of atherosclerosis induced by the progression of CVD. Notably, caution is needed in interpreting the biological mechanisms of these associations because, to date, potentially involved compounds and modes of action are diverse and evidence is still limited.

In our study, the concept and topic of UPF that first appeared in 2009 were relatively new to British people.^26^ As a prospective study, the UK Biobank did not provide participants with any dietary recommendations but only collected information about their diet.^38^ The probability of dietary modifications is low, and UPF consumption remains stable^7^ within the timeframe considered in this study. Therefore, we believe that participants are likely not to modify their dietary behaviours. Moreover, secondary models focussing on participants whose proportion of UPF in their diet varied by <|0.2| provided similar results between the beginning and the progression of their follow-up. Our research builds on the large prospective population-based UK Biobank using the most frequently used method (NOVA framework) to identify and categorize UPFs.

However, this study also had some limitations. First, owing to incomplete information, some misclassifications in the NOVA category of UPFs cannot be ruled out. This would have resulted in a non-differential measurement error and potential bias towards the null hypothesis. Second, due to the nature of the study, it was not possible to use validated questionnaires^25^ to calculate UPF consumption. However, UPF consumption calculated from the 24-h dietary recall questionnaire was used in other cohort studies.^10^^,^^16^^,^^23^ Third, although case ascertainment through electronic health record data allowed us to maximize case detection, exhaustiveness could not be guaranteed. The resulting potential misclassification bias was likely to be undifferentiated owing to the prospective design. Further research is needed to examine the use of multiple coded data sources to improve accuracy and completeness. Lastly, the length of follow-up was relatively limited as the UK Biobank cohort was launched in March 2006. This allowed us to investigate mostly mid-term associations between the consumption of UPFs and the risk of CVD.

As cardiovascular event processes may take several decades, it will be the next work to reassess the associations between UPF and CVD risk in the future. Fourthly, using only the baseline 24-h dietary recall questionnaire is a limitation, but it was reported as representative dietary. Lastly, among those invited to participate in the UK Biobank cohort, 5.5% of participants only gave a response in the baseline assessment. Participants were more likely to be female, older, lived in less socioeconomically deprived areas than non-participants, less likely to be obese, and had fewer self-reported health conditions than the general population.^39^ This might have underestimated the associations owing to a lower range of UPF intake. Furthermore, this selection bias did not seem to affect the actual association for the large sample size. The effect sizes observed in this study were consistent with other large nutritional epidemiological cohorts.^12^^,^^14^^,^^23^^,^^24^

Although the HRs of UPF exposure appeared to be relatively limited, the direct and indirect public health effects of these associations could be vital because UPF consumption is widespread in the general population. Policy-based strategies for healthy behaviour are likely to be more effective than individual-level interventions for achieving sustained changes in lifestyle.^28^ Limiting UPF intake in the diet is more acceptable and easier to implement than previously accepted nutrient intake recommendations. Public health efforts should focus on a broader concept of consumption decisions (e.g. choosing healthy foods), such as increasing access to minimally processed food and dishes that are convenient, tasty and affordable. Reducing UPF consumption requires simultaneous efforts to advance supply and demand at local, national and transnational levels. Further epidemiological and experimental studies are required to elucidate the underlying mechanisms for the association of UPF consumption with CVD risk and all-cause mortality.

In conclusion, UPF consumption is associated with CVD, CHD, cerebrovascular disease and all-cause mortality in the UK Biobank cohort. These findings support existing evidence on the negative impact of UPFs on CVD and all-cause mortality and highlight the importance of minimizing UPF intake for preventing CVD and all-cause mortality.

## Ethics approval and consent to participate

The current study was conducted in accordance with the guidelines of the Declaration of Helsinki, and all procedures involving human subjects were approved by the UK Biobank Ethics and Governance Council (UK Biobank Resource under Application Number 68136). The UK Biobank was approved by the Northwest Multicentre Research Ethics Committee (16/NW/0274). Written informed consent was obtained from all the subjects.

## Supplementary data

Supplementary data are available at *EURPUB* online.

## Supplementary Material

ckac104_Supplementary_Data
