# Supplementary material for: Associations of ultra-processed food consumption with cardiovascular disease and all-cause mortality: UK Biobank
Source: Eur J Public Health. 2022 Aug 25;32(5):779–85. doi: 10.1093/eurpub/ckac104 (PMC9527958; doi:10.1093/eurpub/ckac104)
Supplement: ckac104_Supplementary_Data [file ckac104_supplementary_data.zip › ejph-2022-05-om-0276-File009.docx]

**Associations of** **ultra-processed food consumption with** **cardiovascular disease and** **all-cause mortality: UK Biobank**

Xuanli Chen^a^, Jiadong Chu^a^, Wei Hu^a^, Na Sun^a^, Qida He^a^, Siyuan Liu^a^, Zhaolong Feng^a^, Tongxing Li^a^, Qiang Han^a^, and Yueping Shen^a^.

^a^Department of Epidemiology and Biostatistics, School of Public Health, Medical College of Soochow University, Suzhou 215123, China;

Correspondence to: Yueping Shen, professor, Department of Epidemiology and Biostatistics, School of Public Health, Medical College of Soochow University, no.199, Renai street, Industrial Park, Suzhou 215123, China. Tel: +86 139 6214 8023; fax: +86 0512 6588 3323; E-mail: [shenyueping@suda.edu.cn](mailto:shenyueping@suda.edu.cn)

**Supplementary Table 1.** The ultra-processed food assessment according to NOVA classification system in UK Biobank.

| Items | **Ultra-processed food** |
| --- | --- |
| Drink | Low calorie drink, fizzy drink, squash, orange juice, grapefruit juice, instant coffee, other coffee^a^, flavored milk, low calorie hot chocolate, hot chocolate, spirits, other alcohol^a^. |
| Cereal | Porridge^a^, oat crunch, sweetened cereal, other cereal^a^, sliced bread^a^, bap^a^, crispbread, snackpot. |
| Sweets | Yogurt^a^, ice-cream, milk-based pudding^a^, other milk-based pudding, soya dessert, fruitcake, cake, doughnut, sponge pudding, cheesecake, other dessert^a^, chocolate bar, white chocolate, milk chocolate, dark chocolate, chocolate-covered raisin, chocolate sweet, diet sweet, sweets, chocolate-covered biscuits, chocolate biscuits, sweet biscuits, cereal bar, other sweets^a^. |
| Snacks | Crisp, savoury biscuits, cheesy biscuits, other savoury snack, powdered/instant soup, canned soup, sausage, crumbed or deep-fried poultry, butter^a^, margarine^a^. |

^a^. Owing to incomplete information of this food item in the UK Biobank, this energy calculation of food item had been weighted as appropriate.

**Supplementary Table 2.** The Resource and Definition of the Selected Covariates.

| **Covariates** | **UK Biobank field ID** | **description** | **type** | **category** |
| --- | --- | --- | --- | --- |
| Ultra-processed food | 100090 | Ultra-processed food was calculated using a 24-h dietary recall questionnaire. We derived ultra-processed food intake level as four groups. | Categorical | first quartile;  between first and second quartile;  between second and third quartile;  highest quartile; |
| Educational years | 6138 | We derived three groups based on 'qualifications' (6138)^1^. | Categorical | Less than 10 years;  between 10 years and 18 years; more than 18 years |
| Age | 21022 | The existing variable 'age at recruitment' (21022) was applied. | Continuous | - |
| Sex | 31 | The existing variable 'sex' (31) was applied. | Categorical | Male;  female |
| BMI | 21001 | The existing variable 'body mass index (BMI)' (21001) was applied^2^. | Categorical | Underweight, <18.5 kg/m^2^;  normal weight, 18.5-25 kg/m^2^;  overweight, 25-30 kg/m^2^;  obese, ≥30 kg/m^2^ |
| Ethnicity | 21000 | The existing variable 'ethnic background' (21000) was applied. | Categorical | White;  others. |
| Sleep duration | 1160 | The existing variable 'sleep duration ' (1160) was applied^3^. | Categorical | Less than 7 h;  between 7 h and 8 h;  more than 8 hours |
| Smoking status | 20116 | The existing variable 'smoking status' (20116) was applied. | Categorical | Never;  previous smoker;  current smoker |
| Socioeconomic status | 189 | The existing variable 'Townsend deprivation index at recruitment' (189) was applied^4^. | Categorical | Low (below lower tertile);  high (higher than or equal to the lower tertile). |
| Physical activity level | 22040 | The existing variable 'summed MET minutes per week for all activity ' (22040) was applied. | Categorical | Less than the lower tertile;  between the lower tertile and upper tertile;  more than the upper tertile;  missing |
| Energy intake |  |  |  |  |
| Total energy | 100002 | The existing variable 'energy' (100002) was applied. | Continuous | **-** |
| Protein | 100003 | The existing variable 'protein' (100003) was applied. | Continuous | **-** |
| Total fat | 100004 | The existing variable 'fat' (100004) was applied. | Continuous | **-** |
| Carbohydrates | 100005 | The existing variable 'carbohydrates' (100005) was applied. | Continuous | **-** |
| Alcohol | 100022 | The existing variable 'alcohol' (100022) was applied. | Continuous | **-** |
| Fibre | 100009 | The existing variable 'englyst dietary fibre' (100005) was applied. | Continuous | **-** |
| Saturated fat | 100006 | The existing variable 'saturated fat' (100006) was applied. | Continuous | **-** |
| Polyunsaturated fat | 100007 | The existing variable 'polyunsaturated fat' (100007) was applied. | Continuous | **-** |
| Trans-fat | 100090 | Trans-fat intakes were calculated using a 24-h dietary recall questionnaire. | Continuous | **-** |
| Hypertension | 131286,6177, 4080, 93, 4079, 94 | Hypertension was defined as a systolic blood pressure (4080, 93) of at least 140 mmHg, diastolic blood pressure (4079, 94) of at least 90 mmHg, self-reported history of hypertension (131286), or use of antihypertensive medication (6177)^5^. | Categorical | Yes;  no |
| Diabetes | 130708,30740, 6177 | Diabetes was defined as fasting blood glucose at least 7.0 mmol/L (30740), a self-reported history of diabetes (130708), or hypoglycaemic treatment (6177)^6^. | Categorical | Yes;  no |
| Dyslipidaemia | 30870, 30780, 30760, 30690,  6177 | Dyslipidaemia was defined as total cholesterol (30690) at least 6.20 mmol/L, LDL-C (30780) at least 4.13 mmol/L, triglyceride levels (30870) at least 2.25 mmol/L, or HDL-C (30760)) at most 1.03 mmol/L or treatment with cholesterol-lowering medication (6177)^7^. | Categorical | Yes;  no |

**Supplementary Table 3.** Baseline characteristics of participants and non-participants (2008) in UK Biobank.

| Median (low quartile-upper quartile) or mean ± standard deviation or quantity (percentage) | | | |
| --- | --- | --- | --- |
| Variables | participants | non-participants | *P* value^a^ |
| N | 66,090(13.15) | 436,399(86.85) |  |
| Age, years | 55.9±8.2 | 56.6±8.1 | <0.001 |
| Sex (Male) | 28,540(43.18) | 200,574(45.96) | <0.001 |
| Education levels, years |  |  |  |
| ≤10 | 24,371(36.88) | 192,979(44.22) | <0.001 |
| 10 to ≤18 | 11,697 (17.70) | 69,426(15.91) |  |
| >18 | 30,022 (45.43) | 173.995(39.87) |  |
| Ethnicity (White) | 61,932 (93.71) | 413,525(94.76) | <0.001 |
| Townsend index(lower) | 20.186(30.54) | 147,188(33.73) | <0.001 |
| Sleep duration, h |  |  |  |
| <7 | 15,987(24.19) | 107,259(24.58) | <0.001 |
| 7 to ≤8 | 45,908 (69.46) | 294,990(67.60) |  |
| >8 | 4,195(6.35) | 34,151(7.83) |  |
| Physical activity |  |  |  |
| Lower tertiles | 17,185(26.00) | 116,766(26.76) | <0.001 |
| Middle | 19,224(29.09) | 115,142(26.38) |  |
| Upper tertiles | 18,960(28.69) | 115,091(26.37) |  |
| Missing | 10,721(16.22) | 89,401(20.49) |  |
| Smoking status |  |  |  |
| Never | 38,068(57.60) | 238,395(54.63) | <0.001 |
| Ever | 22,457 (33.98) | 150,593(34.51) |  |
| Current | 5,565(8.42) | 47,412(10.86) |  |
| BMI status, kg/m^2^ |  |  |  |
| Underweight (<18.5) | 201(0.30) | 1,292(0.30) | <0.001 |
| Normal weight (18.5 to<25) | 23,925(36.20) | 139,726(32.02) |  |
| Overweight (25 to <30) | 27,475(41.57) | 187,722(43.02) |  |
| Obese (≥30) | 14,489(21.92) | 107,660(24.67) |  |
| Hypertension (yes) | 32,496 (49.17) | 242,728(55.62) | <0.001 |
| Diabetes (yes) | 1,470(2.22) | 14,795(3.39) | <0.001 |
| Dyslipidemia (yes) | 35,137(53.17) | 249,742(57.23) | <0.001 |

^a^: Analysis of variance, Kruskal–Wallis test or χ2 test where appropriate.

**Supplementary Table 4.** Sensitivity analyses excluding outcome within the first 2 years of follow-up (n < 1,000) for the associations between ultra-processed foods intake and risk of CVD and mortality according to consumption of ultra-processed foods (2008-2020) in UK Biobank.

| **Outcome** | Group 1 | Group 2 | Group 3 | Group 4 | *P* trend |
| --- | --- | --- | --- | --- | --- |
| **CVD** |  |  |  |  |  |
| Cases^a^ [n (%)] | 1,181(7.94) | 1,282(8.61) | 1,341(9.02) | 1,443(9.70) |  |
| Person-year incidence | 7.48‰ | 8.12‰ | 8.52‰ | 9.19‰ |  |
| Model 3^b^ | 1(ref) | 1.06(0.97-1.14) | 1.10(1.01-1.20) | 1.20(1.09-1.31) | <0.001 |
| **CHD** |  |  |  |  |  |
| Cases^a^ [n (%)] | 1,033(6.87) | 1,129(7.49) | 1,190(7.91) | 1,258(8.36) |  |
| Person-year incidence | 6.44‰ | 7.03‰ | 7.43‰ | 7.88‰ |  |
| Model 3^b^ | 1(ref) | 1.05(0.97-1.15) | 1.11(1.02-1.21) | 1.17(1.06-1.29) | <0.001 |
| **Cerebrovascular diseases** |  |  |  |  |  |
| Cases^a^ [n (%)] | 303(1.91) | 338(2.13) | 320(2.02) | 394(2.48) |  |
| Person-year incidence | 1.76‰ | 1.96‰ | 1.85‰ | 2.28‰ |  |
| Model 3^b^ | 1(ref) | 1.11(0.95-1.30) | 1.06(0.91-1.25) | 1.32(1.14-1.54) | <0.001 |
| **All-cause mortality** |  |  |  |  |  |
| Cases^a^ [n (%)] | 546(3.40) | 586(3.64) | 577(3.59) | 699(4.35) |  |
| Person-year incidence | 3.14‰ | 3.36‰ | 3.32‰ | 4.02‰ |  |
| Model 3^b^ | 1(ref) | 1.06(0.95-1.20) | 1.04(0.92-1.18) | 1.25(1.09-1.42) | <0.001 |
| **CVD mortality** |  |  |  |  |  |
| Cases^a^ [n (%)] | 71(0.44) | 89(0.55) | 95(0.59) | 92(0.57) |  |
| Person-year incidence | 0.40‰ | 0.50‰ | 0.54‰ | 0.52‰ |  |
| Model 3^b^ | 1(ref) | 1.18(0.86-1.63) | 1.22(0.88-1.70) | 1.10(0.76-1.58) | 0.336 |

Values were presented as hazard ratios (95% confidence interval).

Group 1 is the proportion UPFs energy of total energy in Quartile 1; Group 2 is the proportion UPFs energy of total energy Quartile 2; Group 3 is the proportion UPFs energy of total energy in Quartile 3; Group 4 is the proportion UPFs energy of total energy in Quartile 4. The cut-off values for quarters of ultra-processed food consumption were 20.8%, 31.3%, 43.0% for all participants.

^a^ Participants in cycle0.

^b^ Adjusted for age, sex, ethnicity, education years, smoking status, Townsend deprivation index, obesity status, sleep duration, total energy, protein, total fat, carbohydrates, alcohol, fiber, saturated fat, polyunsaturated fat and trans-fat intake, physical activity, hypertension, dyslipidemia and diabetes.

**Supplementary Table 5.** Sensitivity analyses including participants take in diet survey in cycle 0, 1, 2 ,3 or 4 for the associations between ultra-processed foods intake and risk of CVD and mortality according to consumption of ultra-processed foods (2008-2020) in UK Biobank.

| **Outcome** | Group 1 | Group 2 | Group 3 | Group 4 | *P* trend |
| --- | --- | --- | --- | --- | --- |
| **CVD** |  |  |  |  |  |
| Cases^a^ [n (%)] | 4,315(9.23) | 4,363(9.71) | 4,614(10.27) | 4,947(11.01) |  |
| Person-year incidence | 8.31‰ | 8.71‰ | 9.24‰ | 9.95‰ |  |
| Model 3^b^ | 1(ref) | 0.97(0.90-1.04) | 1.06(0.99-1.14) | 1.11(1.03-1.20) | <0.001 |
| **CHD** |  |  |  |  |  |
| Cases^a^ [n (%)] | 3,767(7.99) | 3,846(8.48) | 4,101(9.04) | 4,402(9.68) |  |
| Person-year incidence | 7.15‰ | 7.57‰ | 8.09‰ | 8.70‰ |  |
| Model 3^b^ | 1(ref) | 0.98(0.90-1.06) | 1.07(0.99-1.16) | 1.12(1.04-1.22) | <0.001 |
| **Cerebrovascular diseases** |  |  |  |  |  |
| Cases^a^ [n (%)] | 1,059(2.15) | 1,042(2.20) | 1,089(2.30) | 1,199(2.53) |  |
| Person-year incidence | 1.87‰ | 1.91‰ | 1.99‰ | 2.20‰ |  |
| Model 3^b^ | 1(ref) | 0.96(0.88-1.05) | 0.99(0.91-1.16) | 1.06(0.97-1.16) | =0.158 |
| **All-cause mortality** |  |  |  |  |  |
| Cases^a^ [n (%)] | 1,718(3.45) | 1,770(3.70) | 1,962(4.10) | 2,188(4.57) |  |
| Person-year incidence | 3.01‰ | 3.21‰ | 3.56‰ | 3.99‰ |  |
| Model 3^b^ | 1(ref) | 1.01(0.94-1.08) | 1.10(1.03-1.18) | 1.19(1.11-1.27) | <0.001 |
| **CVD mortality** |  |  |  |  |  |
| Cases^a^ [n (%)] | 216(0.43) | 259(0.54) | 299(0.62) | 323(0.67) |  |
| Person-year incidence | 0.38‰ | 0.47‰ | 0.54‰ | 0.58‰ |  |
| Model 3^b^ | 1(ref) | 1.14(0.95-1.37) | 1.27(1.06-1.52) | 1.28(1.06-1.54) | 0.004 |

Values were presented as hazard ratios (95% confidence interval).

Group 1 is the proportion UPFs energy of total energy in Quartile 1; Group 2 is the proportion UPFs energy of total energy Quartile 2; Group 3 is the proportion UPFs energy of total energy in Quartile 3; Group 4 is the proportion UPFs energy of total energy in Quartile 4. The cut-off values for quarters of ultra-processed food consumption were 20.8%, 31.3%, 43.0% for all participants.

^a^ Participants in cycle0,1,2,3,4.

^b^ Adjusted for age, sex, ethnicity, education years, smoking status, Townsend deprivation index, obesity status, sleep duration, total energy, protein, total fat, carbohydrates, alcohol, fiber, saturated fat, polyunsaturated fat and trans-fat intake, physical activity, hypertension, dyslipidemia and diabetes.

**Supplementary Table 6.** Sensitivity analyses including participants whose ultra-processed foods intake varied by less than |0.2| survey in cycle 0, 1, 2, 3 or 4 for the associations between UPFs and risk of CVD and mortality according to consumption of ultra-processed foods (2008-2020) in UK Biobank.

| **Outcome** | Group 1 | Group 2 | Group 3 | Group 4 | *P* trend |
| --- | --- | --- | --- | --- | --- |
| **CVD** |  |  |  |  |  |
| Cases^a^ [n (%)] | 1,158(8.58) | 1,145(8.95) | 1,202(9.40) | 1,356(10.60) |  |
| Person-year incidence | 7.64‰ | 7.96‰ | 8.36‰ | 9.47‰ |  |
| Model 3^b^ | 1(ref) | 1.00(0.92-1.08) | 1.03(0.95-1.12) | 1.13(1.04-1.23) | <0.001 |
| **CHD** |  |  |  |  |  |
| Cases^a^ [n (%)] | 1,035(7.60) | 1,014(7.86) | 1,063(8.26) | 1,208(9.36) |  |
| Person-year incidence | 6.75‰ | 6.95‰ | 7.29‰ | 8.32‰ |  |
| Model 3^b^ | 1(ref) | 0.98(0.90-1.07) | 1.01(0.93-1.10) | 1.12(1.03-1.22) | <0.001 |
| **Cerebrovascular diseases** |  |  |  |  |  |
| Cases^a^ [n (%)] | 257(1.81) | 258(1.92) | 297(2.14) | 322(2.40) |  |
| Person-year incidence | 1.57‰ | 1.65‰ | 1.90‰ | 2.06‰ |  |
| Model 3^b^ | 1(ref) | 1.01(0.85-1.21) | 1.13(0.95-1.34) | 1.24(1.05-1.47) | <0.001 |
| **All-cause mortality** |  |  |  |  |  |
| Cases^a^ [n (%)] | 437(3.05) | 460(3.39) | 519(3.83) | 523(3.86) |  |
| Person-year incidence | 2.64‰ | 2.92‰ | 3.30‰ | 3.32‰ |  |
| Model 3^b^ | 1(ref) | 1.06(0.93-1.20) | 1.19(1.05-1.35) | 1.16(1.02-1.32) | <0.001 |
| **CVD mortality** |  |  |  |  |  |
| Cases^a^ [n (%)] | 63(0.44) | 59(0.44) | 70(0.52) | 79(0.58) |  |
| Person-year incidence | 0.38‰ | 0.37‰ | 0.44‰ | 0.50‰ |  |
| Model 3^b^ | 1(ref) | 0.94(0.66-1.35) | 1.11(0.79-1.57) | 1.20(0.85-1.68) | 0.530 |

Values were presented as hazard ratios (95% confidence interval).

Group 1 is the proportion UPFs energy of total energy in Quartile 1; Group 2 is the proportion UPFs energy of total energy Quartile 2; Group 3 is the proportion UPFs energy of total energy in Quartile 3; Group 4 is the proportion UPFs energy of total energy in Quartile 4. The cut-off values for quarters of ultra-processed food consumption were 20.8%, 31.3%, 43.0% for all participants.

^a^ Participants in cycle0,1,2,3,4.

^b^ Adjusted for age, sex, ethnicity, education years, smoking status, Townsend deprivation index, obesity status, sleep duration, total energy, protein, total fat, carbohydrates, alcohol, fiber, saturated fat, polyunsaturated fat and trans-fat intake, physical activity, hypertension, dyslipidemia and diabetes.


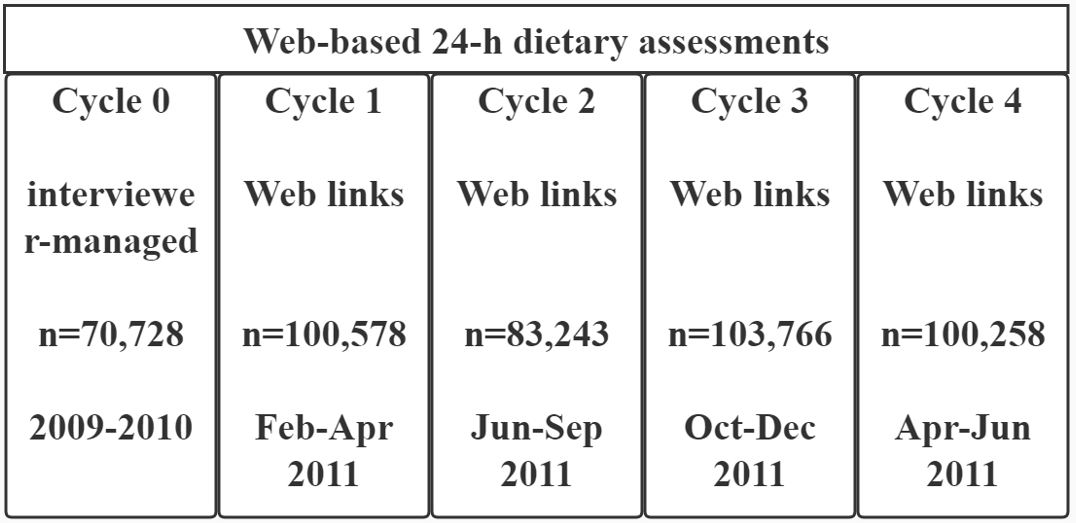


**Supplementary Figure 1.** Number of UK Biobank participants who completed 24-hour dietary recall questionnaire at recruitment and web-based 24-h dietary assessments.


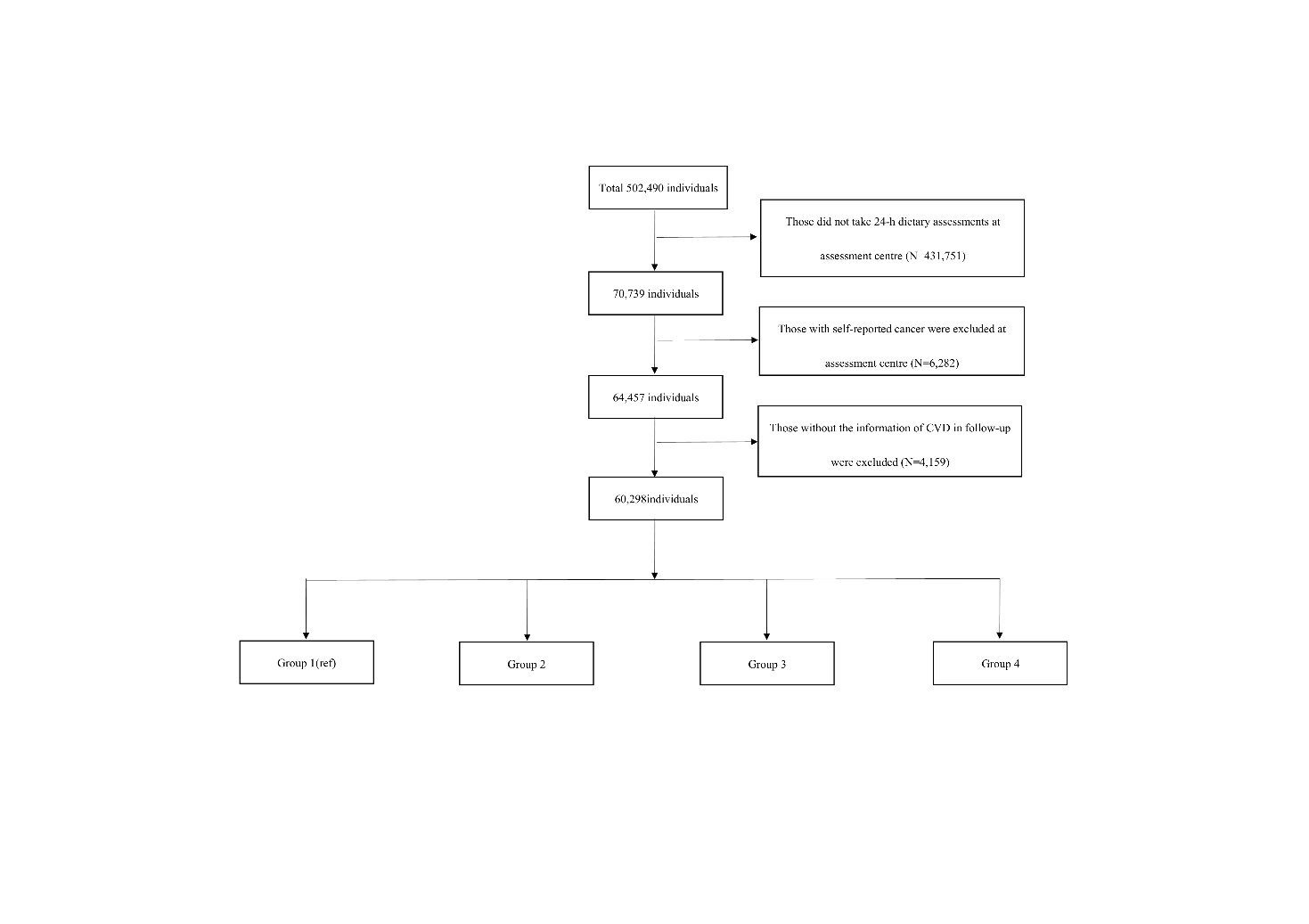


**Supplementary Figure 2.** Flowchart of study participants according to inclusion and exclusion criteria in UK Biobank at baseline.


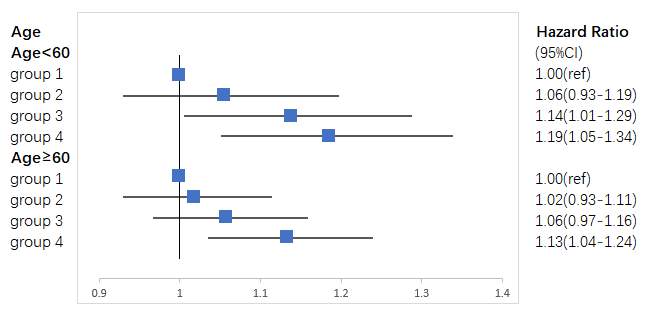

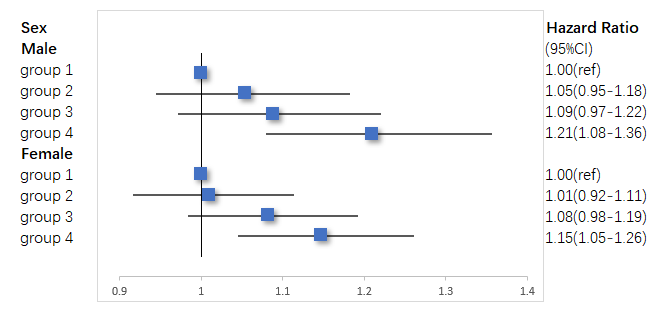


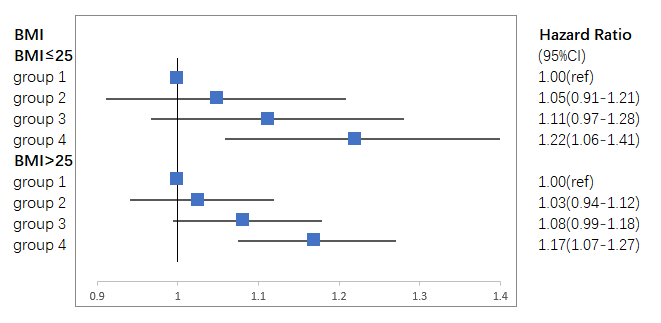

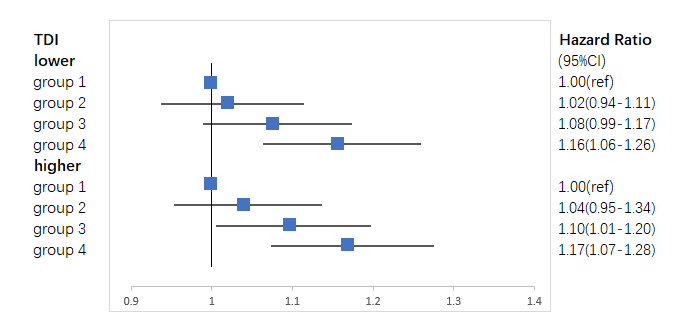


**Supplementary Figure 3.** The association between consumption ultra-processed foods and CVD in different subgroups according to consumption ultra-processed foods (2008-2020) in UK Biobank.

**Reference**

1 Cheesman R, Coleman J, Rayner C, et al. Familial Influences on Neuroticism and Education in the UK Biobank. Behav Genet 2020;50:84-93.

2 Renehan AG, Tyson M, Egger M, Heller RF, Zwahlen M. Body-mass index and incidence of cancer: a systematic review and meta-analysis of prospective observational studies. The Lancet 2008;371:569-578.

3 Huang BH, Duncan MJ, Cistulli PA, Nassar N, Hamer M, Stamatakis E. Sleep and physical activity in relation to all-cause, cardiovascular disease and cancer mortality risk. Br J Sports Med 2021.

4 Pei YF, Zhang L. Is the Townsend Deprivation Index a Reliable Predictor of Psychiatric Disorders? Biol Psychiatry 2021;89:839-841.

5 Mills KT, Stefanescu A, He J. The global epidemiology of hypertension. Nat Rev Nephrol 2020;16:223-237.

6 Chatterjee S, Khunti K, Davies MJ. Type 2 diabetes. The Lancet 2017;389:2239-2251.

7 Kopin L, Lowenstein C. Dyslipidemia. Ann Intern Med 2017;167:ITC81-ITC96.
