# Supplementary figures and images for: Associations of ultra-processed food consumption with cardiovascular disease and all-cause mortality: UK Biobank
Source: Eur J Public Health. 2022 Aug 25;32(5):779–85. doi: 10.1093/eurpub/ckac104 (PMC9527958; doi:10.1093/eurpub/ckac104)

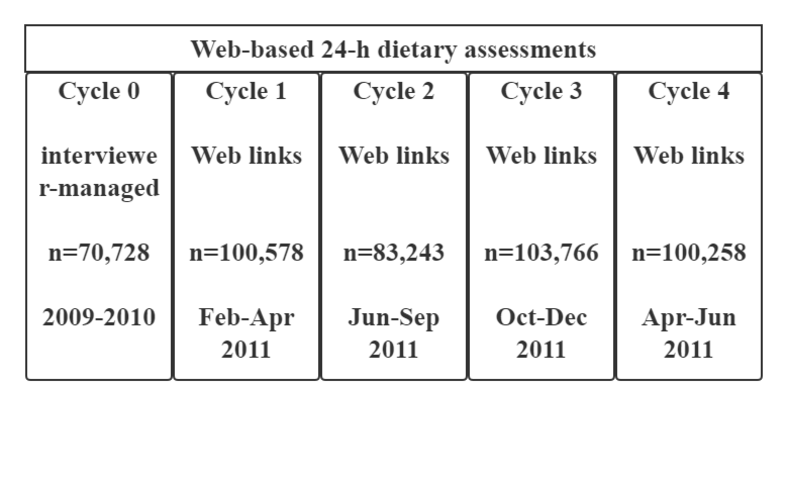

Supplement: ckac104_Supplementary_Data [file ckac104_supplementary_data.zip › ejph-2022-05-om-0276-File010.tif]

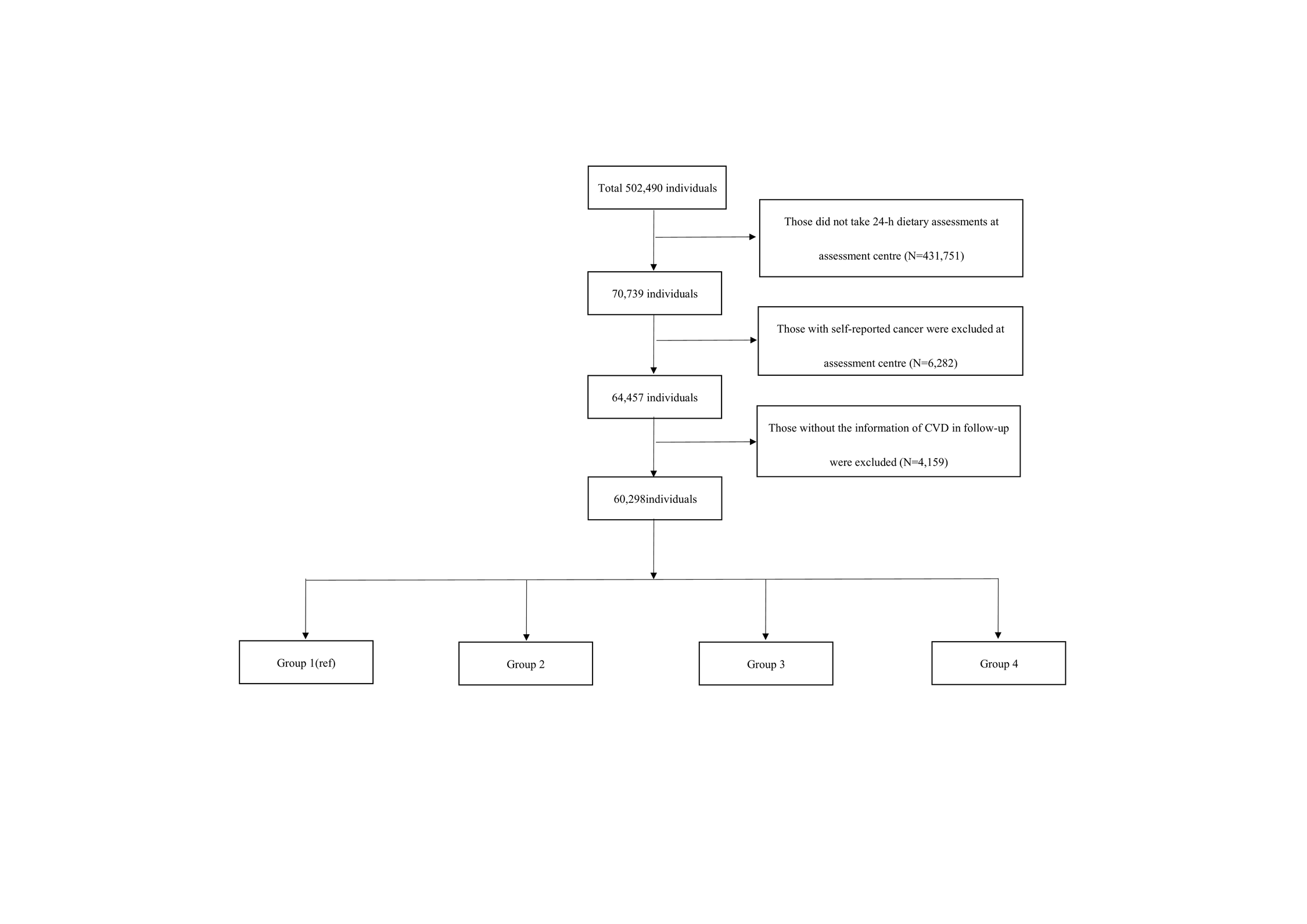

Supplement: ckac104_Supplementary_Data [file ckac104_supplementary_data.zip › ejph-2022-05-om-0276-File011.tif]

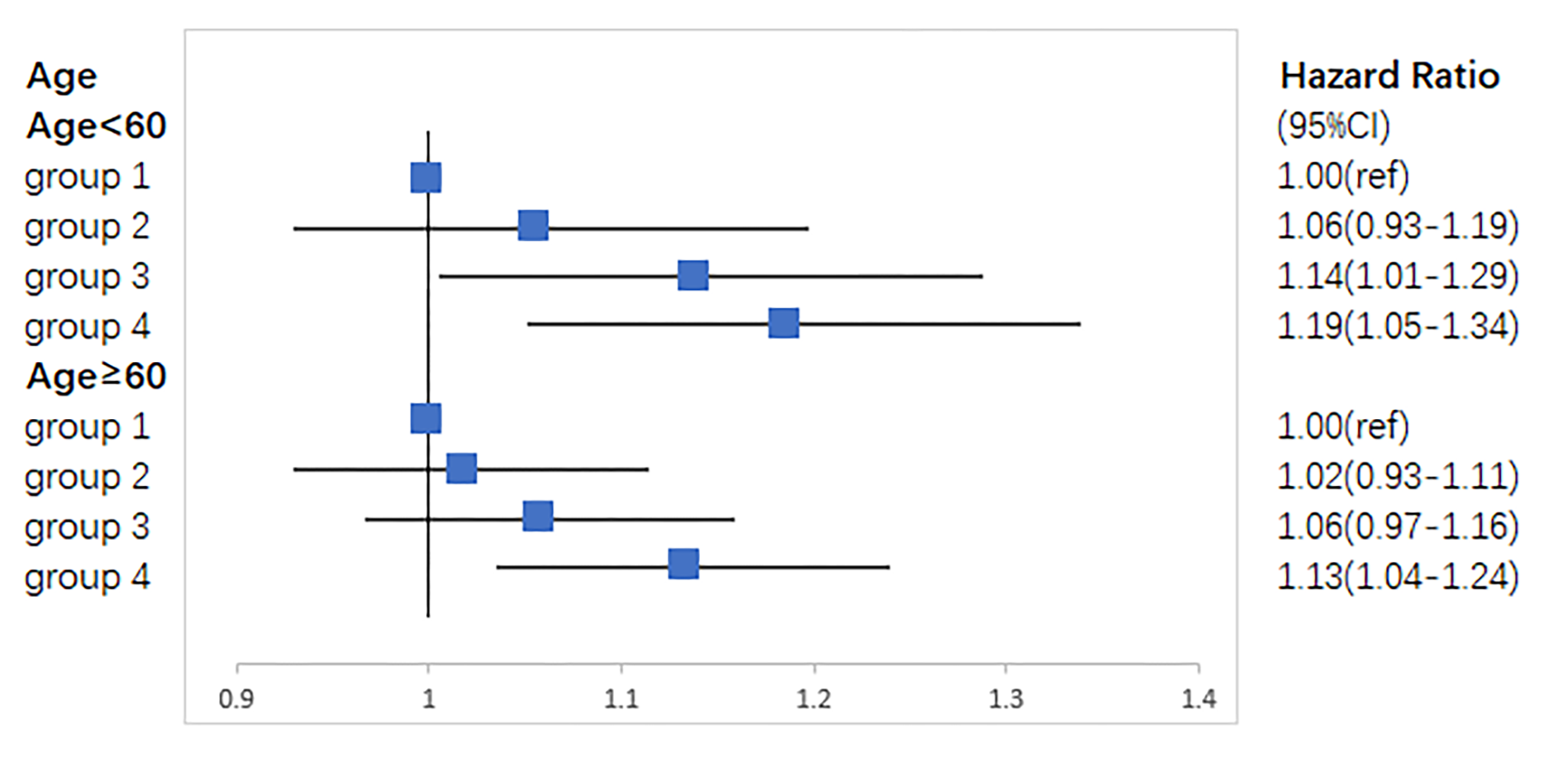

Supplement: ckac104_Supplementary_Data [file ckac104_supplementary_data.zip › ejph-2022-05-om-0276-File012.tif]

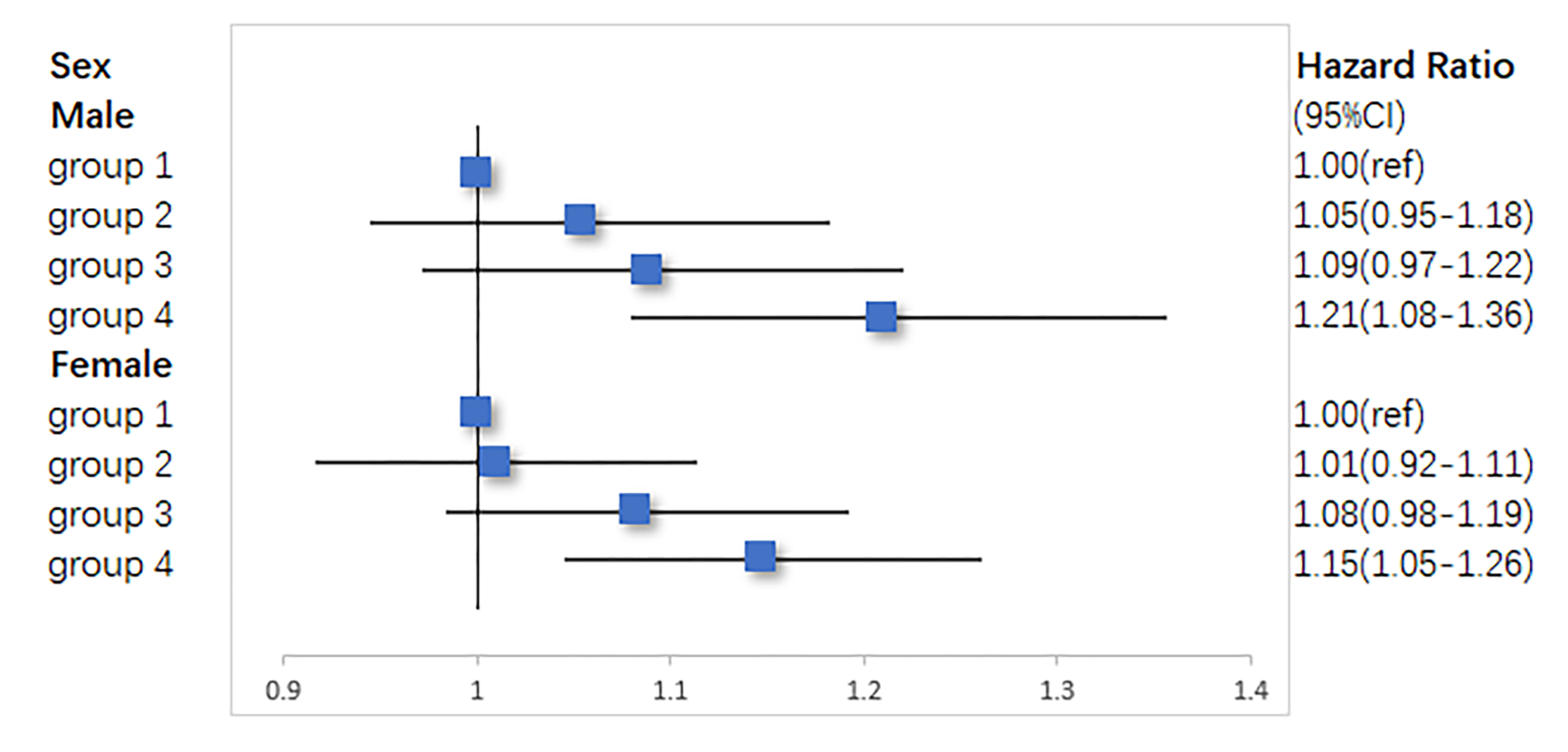

Supplement: ckac104_Supplementary_Data [file ckac104_supplementary_data.zip › ejph-2022-05-om-0276-File013.tif]

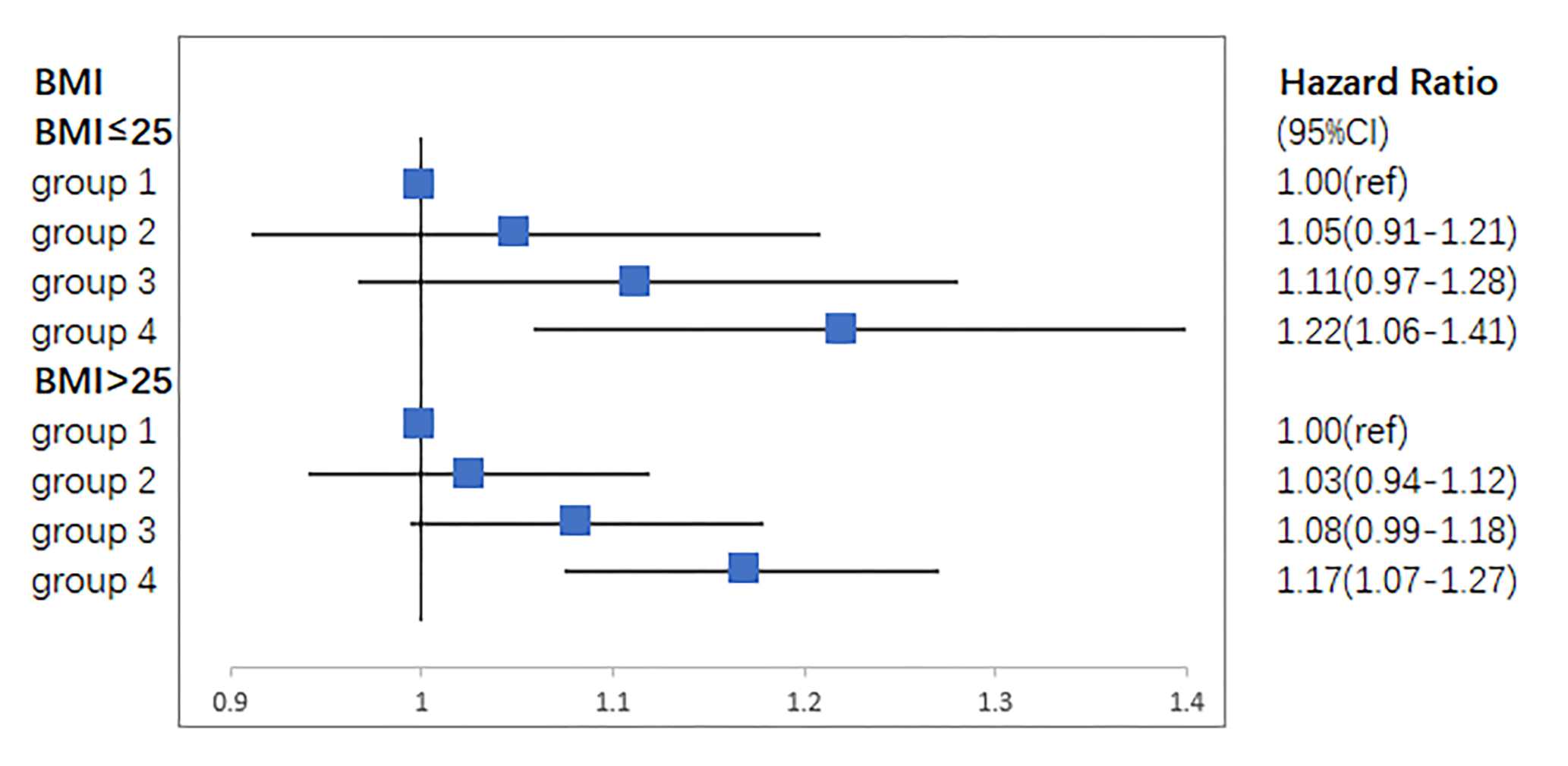

Supplement: ckac104_Supplementary_Data [file ckac104_supplementary_data.zip › ejph-2022-05-om-0276-File014.tif]

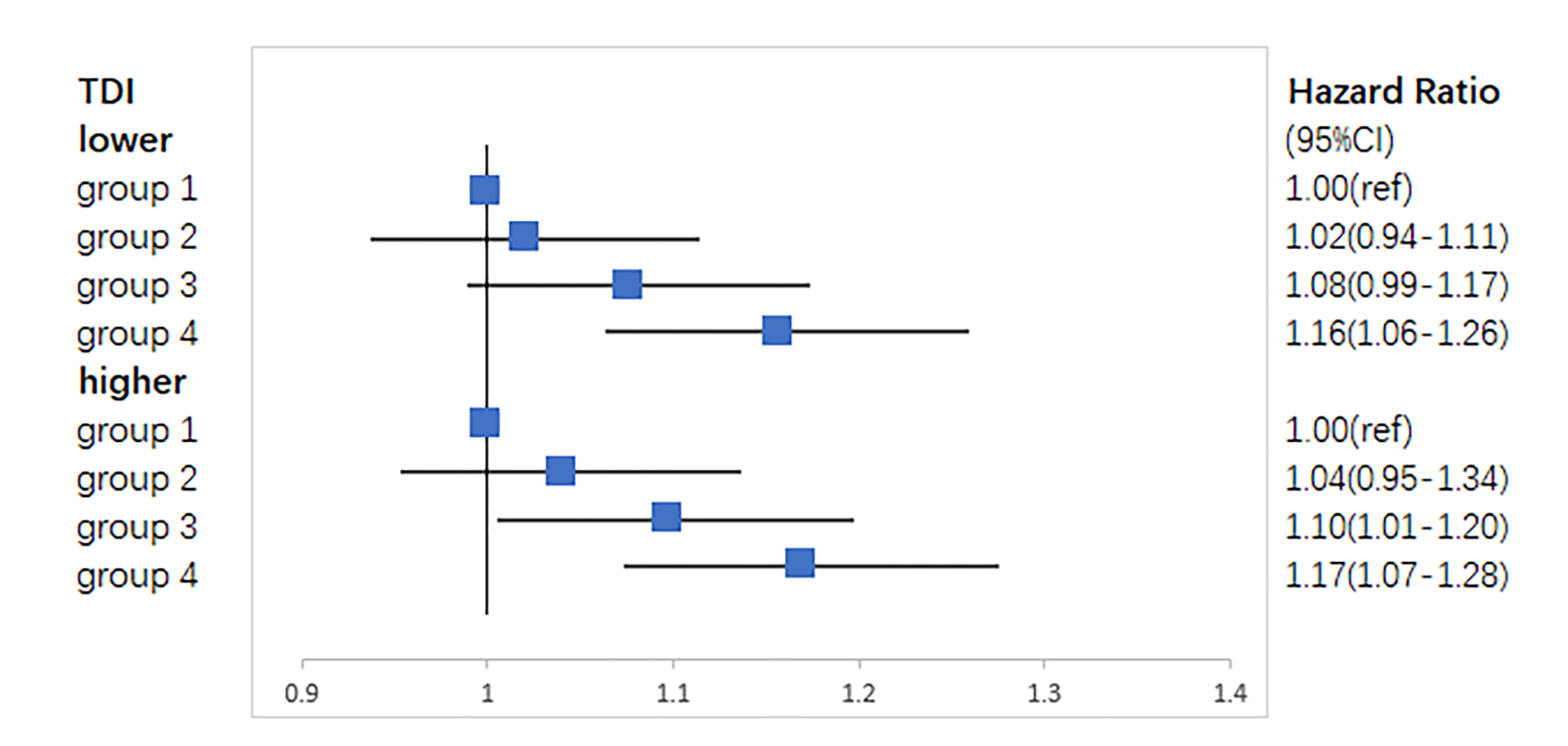

Supplement: ckac104_Supplementary_Data [file ckac104_supplementary_data.zip › ejph-2022-05-om-0276-File015.tif]
